# Case Report: Rhabdomyolysis following initiation of tirzepatide

**DOI:** 10.3389/fphar.2025.1660785

**Published:** 2025-08-20

**Authors:** Jonas Michael Bodanowitz, Isabell Mattes, Micha Loebermann, Carlos Fritzsche

**Affiliations:** Department of Internal Medicine, University Medicine, Rostock, Germany

**Keywords:** tirzepatide, rhabdomyolysis, GLP-1 receptor agonist, adverse drug reaction, muscle injury

## Abstract

We present the case of a 66-year-old woman who developed weakness, nausea, and vomiting accompanied by markedly elevated creatine kinase levels after first treatment with an increased dose of tirzepatide. Laboratory findings were consistent with rhabdomyolysis and normalized within 4 days following discontinuation of tirzepatide and initiation of supportive intravenous fluid therapy. The temporal relationship strongly suggests tirzepatide as a likely trigger. Off-label use, particularly for weight loss, should be avoided and approached with caution. To the best of our knowledge, this is the first reported case of rhabdomyolysis following the initial administration of tirzepatide.

## 1 Introduction

Rhabdomyolysis is characterized by the breakdown of skeletal muscle fibers, resulting in the release of intracellular contents such as myoglobin and creatine kinase (CK) into the bloodstream (Cabral et al., 2020). The clinical presentation of this condition varies widely, ranging from asymptomatic elevations in muscle enzymes to severe complications, including electrolyte disturbances, acute kidney injury, and metabolic derangements (Cabral et al., 2020; Melli et al., 2005). Notably, up to 50% of cases may remain clinically silent (Cervellin et al., 2010). Drug-induced rhabdomyolysis is a well-recognized phenomenon, most associated with statin therapy (Montastruc, 2023). A literature search revealed two cases of rhabdomyolysis subsequent to glucagon-like peptide-1 (GLP-1) receptor agonist treatment. One case involved semaglutide and another case involved tirzepatide (Billings et al., 2023; Sonavane et al., 2025). Interestingly, in both cases GLP-1 receptor agonist treatment was administered for the purpose of weight reduction. In the case of tirzepatide muscle injury developed several months after treatment was started. Given tirzepatide’s increasing use in both diabetes management and weight reduction, the recognition and reporting of rare adverse events are essential. Rhabdomyolysis has not been reported in clinical trials leading to the approval of tirzepatide.

## 2 Case presentation

A 66-year-old woman presented to the emergency department with profound weakness, nausea, and repeated vomiting, 1 day after receiving her first subcutaneous dose of tirzepatide (Mounjaro®) at a supratherapeutic dose of 15 mg (recommended starting dose: 2.5 mg). The patient reported she did not adhere to the information in the package leaflet and used a higher dose to achieve faster weight loss.

The patient’s past medical history included neuropathic pain and hyperlipoproteinemia. Her current medications included atorvastatin, gabapentin, and amitriptyline had remained unchanged for over 3 years and were paused during the inpatient stay. Physical examination revealed no abnormalities. The patient denied myalgia, and neurological assessment showed preserved muscle strength. Initial laboratory investigations, performed approximately 24 h after tirzepatide administration, were within normal limits.

**FIGURE 1:**
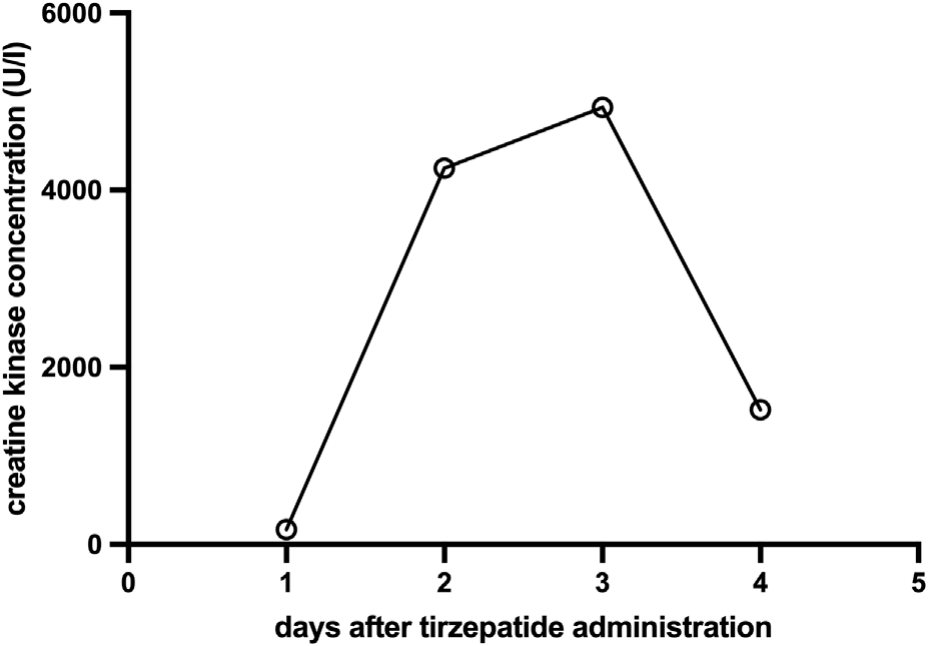
Evolution of creatine kinase concentration (U/L) over time (days) after a single subcutaneous dose of 15 mg tirzepatide on day 0 (creatine kinase reference <170 U/L).

However, follow-up blood tests obtained approximately 48 h after tirzepatide injection revealed significant elevations in the following laboratory parameters: creatine kinase (CK) 4249 U/L (reference <170 U/L), myoglobin 472 ng/mL (reference 25–58 ng/mL), AST 87.5 U/L (reference <35 U/L), and ALT 37.4 U/L (reference <35 U/L) (Figure 1; Table 1). Renal function and inflammatory markers remained within normal limits. Chest X-ray and abdominal ultrasound showed no pathological findings. Further laboratory investigations revealed no evidence of active viral hepatitis. Tests for *leptospira*, Epstein–Barr virus (EBV), and cytomegalovirus (CMV) infection were negative. No bacterial or viral pathogens were detected in the throat swab, and there was no indication of a urinary tract infection. The patient was treated with isotonic intravenous crystalloids and antiemetics. Her symptoms resolved, CK levels declined (Figure 1), and all abnormal laboratory values normalized within 3 days. The clinical course strongly implicated tirzepatide as the causative agent. During an outpatient follow-up visit 8 weeks after discharge, the patient reported no complaints and routine blood tests were within normal limits.

**TABLE 1:** Overview of all laboratory parameters following tirzepatide administration on day 0 (abnormal values are indicated in bold).

Parameter	Reference	day1	day2	day3	day4
Hemoglobin	7.4–9.9 mmol/L	8.8 mmol/L	8.6 mmol/L	7.8 mmol/L	8.1 mmol/L
Hematocrit	0.35–0.47	0.43	0.42	0.36	0.39
Platelets	150–400 × 10^9^/l	194 × 10^9^/l	205 × 10^9^/l	152 × 10^9^/l	**145 x10** ^**9**^ **/l (−)**
White blood cell count	4–9 x10^9^/l	**12.5 x10** ^**9**^ **/l (+)**	**15.1 x10** ^**9**^ **/l (+)**	**9.29 x10** ^**9**^ **/l (+)**	**10.0 x10** ^**9**^ **/l (+)**
Sodium	136–145 mmol/L	143 mmol/L	143 mmol/L	141 mmol/L	136 mmol/L
Potassium	3.4–4.5 mmol/L	3.8 mmol/L	4.4 mmol/L	4.0 mmol/L	**3.0 mmol/L (−)**
Calcium	2.20–2.35 mmol/L	2.35 mmol/L	2.40 mmol/L	2.26 mmol/L	**2.15 mmol/L (−)**
Phosphate	0.81–1.45 mmol/L	Not measured	Not measured	Not measured	**0.57 mmol/L (−)**
Alanine aminotransferase (ALT)	<35 U/L	23.5 U/L	**37.4 U/L (+)**	**67.6 U/L (+)**	**86.7 U/L (+)**
Aspartate aminotransferase (AST)	<35 U/L	26.5 U/L	**87.5 U/L (+)**	**150 U/L (+)**	**108 U/L (+)**
Alkaline Phosphatase (AP)	35–104 U/L	**116 U/L (+)**	**106 U/L (+)**	94.7 U/L	Not measured
Gamma-glutamyl Transferase	<40 U/L	14.5 U/L	16.9 U/L	28.0 U/L	Not measured
Bilirubin total	<15 μmol/L	6.87 μmol/L	11.2 μmol/L	8.69 μmol/L	Not measured
Lactate dehydrogenase (LDH)	135–214 U/L	214 U/L	**312 U/L (+)**	**312 U/L (+)**	Not measured
Creatine Kinase (CK)	<170 U/L	167 U/L	**4249 U/L (+)**	**4934 U/L (+)**	**1520 U/L (+)**
Myoglobin	25–58 ng/mL	Not measured	Not measured	**472 ng/mL (+)**	**99.2 ng/mL (+)**
Creatinine	45–84 μmol/L	57.4 μmol/L	61.6 μmol/L	60.7 μmol/L	50.1 μmol/L
C-reactive protein (CRP)	<5 mg/L	2.56 mg/L	2.77 mg/L	3.07 mg/L	2.67 mg/L
Procalcitonin (PCT)	<0.046 ng/mL	Not measured	Not measured	0.038 ng/mL	Not measured
Ceruloplasmin	0.2–0.6 g/L	Not measured	Not measured	Not measured	0.243 g/L
Ferritin	13–300 μg/L	Not measured	Not measured	Not measured	207 μg/L
HbA1c	<6.0%	Not measured	Not measured	5.7%	Not measured
Thyroid-stimulating hormone (TSH)	0.27–4.2 µIU/mL	Not measured	Not measured	1.63 µIU/mL	Not measured

## 3 Discussion

To our knowledge, this is the second reported case implicating tirzepatide as a causative agent for rhabdomyolysis. The temporal association between drug administration and symptom onset, along with the exclusion of other potential causes—such as trauma, current infections, electrolyte disturbances, and drug abuse—strongly supports this link. The rapid onset of the typical symptoms such as nausea and vomiting within 24 h of drug initiation, the subsequent development of rhabdomyolysis and clinical improvement following drug discontinuation support a causal relationship.

Although drug-induced rhabdomyolysis is well established—particularly in association with statins—tirzepatide itself was not described as an inducer of rhabdomyolysis (Thomsen et al., 2025). As a dual GLP-1 and GIP receptor agonist, tirzepatide modulates insulin sensitivity, lipid metabolism, and inflammatory pathways (Nauck and Müller, 2023). Its pharmacodynamic effects include delayed gastric emptying and modulation of incretin activity, which may indirectly impact systemic homeostasis and muscle metabolism (Müller et al., 2019). It is conceivable that supratherapeutic activation of GLP-1 and GIP receptors may provoke exaggerated metabolic responses and cellular stress in skeletal muscle tissue, particularly in the presence of additional risk factors such as concomitant statin use. Although this mechanism alone may not fully account for the clinical course, initiation of tirzepatide at a supratherapeutic starting dose of 15 mg subcutaneously may have been a contributing factor. While atorvastatin is known to cause muscle toxicity, the patient had tolerated it for over 3 years without prior complications. Dehydration secondary to tirzepatide induced nausea and vomiting may also have played a role in the development of rhabdomyolysis, as volume depletion can lead to reduced muscle perfusion, electrolyte disturbances, and metabolic stress—all recognized risk factors for myocyte injury (Singhal et al., 1992).

The increasing off-label use of tirzepatide for cosmetic weight loss, particularly in patients without metabolic disease, underscores the need for vigilant post-marketing surveillance. In this case, tirzepatide was prescribed off-label for weight reduction, despite the patient’s BMI being 25.3 kg/m^2^. According to the manufacturer’s guidelines, a BMI of ≥27 kg/m^2^ is required for weight management indications. While rhabdomyolysis has previously been reported after several months of tirzepatide therapy, the present case is notable for the onset of this adverse effect following a single supratherapeutic dose (Sonavane et al., 2025).

In conclusion, the underlying pathophysiological mechanism remains uncertain. While common adverse effects such as gastrointestinal symptoms are well documented (Jastreboff et al., 2022), rare and serious events like rhabdomyolysis may only emerge with broader clinical use. This case underscores the importance of informed consent, individualized risk-benefit assessment, and close monitoring, particularly when tirzepatide is used outside of approved indications.

## Data Availability

The original contributions presented in the study are included in the article/supplementary material, further inquiries can be directed to the corresponding author.
